# A Drive–Vibration Integrated Piezoelectric Actuator for Flexible Electrode Implantation

**DOI:** 10.3390/mi17040447

**Published:** 2026-04-03

**Authors:** Xinhui Li, Di Wu, Xiaohui Lin, Tianyu Jiang, Jijie Ma, Ya Li, Yili Hu, Yingting Wang, Hongbo Zhong, Xinyu Yang, Jianping Li, Jianming Wen

**Affiliations:** 1 The Institute of Precision Machinery and Smart Structure, College of Engineering, Zhejiang Normal University, Jinhua 321004, China; 2 Xingzhi College, Zhejiang Normal University, Jinhua 321004, China; 3 Hangzhou Zhiyuan Research Institute Co., Ltd., No. 188, Yunzhan Road, Xihu District, Hangzhou 310012, China

**Keywords:** piezoelectric actuator, drive–vibration integration, flexible electrode, vibration-assisted implantation

## Abstract

In this paper, a drive–vibration integrated piezoelectric actuator (DVIPA) is proposed for vibration-assisted implantation of flexible electrodes. Conventional implantation systems typically rely on separate actuation and vibration modules, which increase system complexity and limit integration. To address this limitation, the proposed DVIPA integrates driving and vibration functions within a single compact structure by employing two piezoelectric bimorphs for clamping and a piezoelectric stack for combined actuation. A composite excitation waveform, consisting of high-frequency sinusoidal signals superimposed on the rising stage of a low-frequency trapezoidal wave, is applied to simultaneously generate forward motion and vibration. This configuration enables a coupled motion mode that facilitates insertion while reducing the risk of buckling. A prototype of the DVIPA was developed and experimentally evaluated. The results show that vibration-assisted implantation can be achieved under various operating conditions, with independently adjustable driving and vibration parameters. A maximum speed of 328 μm/s is obtained, meeting the requirements for flexible electrode implantation. Agarose gel experiments further demonstrate that vibration frequencies above 40 Hz and voltages between 20 and 40 V can effectively assist implantation of polydimethylsiloxane (PDMS) without buckling failure. Overall, the proposed actuator provides a compact and integrated solution for vibration-assisted implantation, offering potential advantages in applications with limited space.

## 1. Introduction

Neural electrodes are crucial components for brain–computer interface (BCI) signal acquisition. Among them, flexible electrodes made from polymer materials can adapt to the micro-movements of the brain better than rigid electrodes [1], facilitating integration with brain tissue, reducing immune responses, and prolonging the functional lifespan of the electrodes [2,3]. While flexible neural electrodes achieve excellent mechanical compatibility, they still pose implantation challenges: flexible electrodes lack the rigidity needed for implantation, facing risks of buckling and rupture during the process, which can lead to secondary damage [4,5].

To address this issue, some researchers have proposed using rigid external auxiliary implantation tools to guide flexible electrodes deeper into the brain tissue [6,7]. Takashi D. Yoshida Kozai et al. introduced an auxiliary implantation tool with a self-assembled monolayer coating. The relative displacement between the coated shuttle and the polymer was reduced from 100% to (1.0 ± 0.66)% [8]. However, the introduction of auxiliary tools increases the implantation volume, thereby enlarging the wound area, and the retracting drag force can further exacerbate brain tissue damage. Alternatively, other scholars have placed flexible electrodes inside capillary structures [9,10]. Charles M. Lieber et al. developed a controllable injection mesh flexible electrode method, achieving targeted delivery of electrodes to ex vivo brain tissue with a spatial precision of 20 μm [11]. Nevertheless, the subsequent cable connection process remains complex and cumbersome. Another strategy involves altering mechanical properties via fluidic channels [12,13]. S. Rezae et al. proposed a flexible method for neural probes with fluid pressure control in microchannels. By controlling the fluid pressure within the channel, the stiffness of the nerve probe can be dynamically regulated [14]. However, the manufacturing process for microchannel structures is complex and costly, and the expansion and deformation of the electrodes during liquid injection may increase the area of the implantation wound. Some researchers proposed another method that uniformly encapsulates rigid biodegradable coatings on the surface of flexible electrodes, thereby temporarily altering the stiffness of the flexible electrodes [15,16]. Zhuolin Xiang et al. designed a flexible polyimide neural probe embedded with maltose-coated microneedles, which dissolve completely in body fluids within seconds post-implantation. [4]. This method struggles to control the thickness and shape of the coating, and the flexible electrode wrapped by the coating cannot collect signals in real-time, making it challenging to capture neural activity accurately.

Consequently, existing methods heavily rely on temporarily increasing stiffness, which inevitably enlarges the implantation footprint, compromises real-time signal monitoring, and precludes secondary implantation once signal attenuation occurs. Rather than artificially increasing electrode stiffness, an alternative strategy is to reduce the tissue penetration resistance. Inspired by the vibration of a mosquito’s proboscis piercing the skin, vibration assistance has emerged as a promising solution to lower the required insertion force, thereby preventing buckling. However, conventional vibration-assisted implantation systems face a critical bottleneck: they typically rely on a physically separated linear actuator coupled with an external vibrator [17]. This disjointed configuration results in a bulky footprint that severely limits their application in spatially constrained stereotaxic neural surgeries.

The piezoelectric actuator, due to its long stroke and micrometer resolution, has been widely utilized in precision actuation systems [18,19]. Recent advancements in piezoelectric driving technologies have demonstrated remarkable motion capabilities, such as tracking optimally smoothed trajectories to achieve uniform, high-throughput scanning while eliminating abrupt mechanical jerks [20,21]. Moreover, advanced piezoelectric positioners have achieved cross-scale, high-bandwidth actuation with nanometer-level precision in demanding applications [22]. Crucially, for stepping-type mechanisms, innovative designs such as zero-phase-difference flexible biped actuators have been developed to effectively suppress backward motion during actuation [23]. Minimizing this backward retraction is exceptionally vital in delicate biomedical manipulations to prevent tissue drag and secondary damage during surgical insertion. Particularly in the neural engineering field, the specific potential of piezoelectric mechanisms has been explicitly explored. For instance, piezoelectric inchworm actuators, combined with smart materials like magnetorheological elastomers, have been developed to provide a compact, steady, and highly controllable insertion mechanism, demonstrating immense potential for Brain–Computer Interface (BCI) implantation tasks [24]. These properties also make it ideal for applications requiring flexible electrode implantation.

Building upon these intrinsic advantages of piezoelectric mechanisms, the primary innovation of this paper lies in the unprecedented structural and functional integration of the implantation device. Instead of utilizing separate auxiliary components or bulky external vibrators, we propose a Drive–Vibration Integrated Piezoelectric Actuator (DVIPA) that intrinsically merges both the macroscopic forward-driving motion and the microscopic assisting vibration into a single, highly compact actuator unit.

A drive–vibration composite waveform, where high-frequency sine waves are superimposed on the rising edge of a low-frequency trapezoidal wave, serves as the excitation signal for the piezoelectric stack, simultaneously driving the flexible electrode forward and generating localized vibrations. This paper presents the structure and principle of the proposed DVIPA, conducts a simulation analysis of the flexure hinge, and fabricates a prototype to test its performance. Finally, implantation experiments are carried out, demonstrating that the compact actuator can effectively and independently assist in the implantation of flexible electrodes.

## 2. Structure and Principle

### 2.1. Structure Design

Different from a rigid neural electrode, flexible electrode implantation suffers from low stiffness of the electrode. Structure design of DVIPA should give full consideration of the following requirements.

Firstly, during the implantation of flexible electrodes, the DVIPA should apply vibration along the implantation direction to reduce implantation damage and assist in electrode insertion. To achieve this, an additional sine wave should be superimposed on the drive signal of the original actuator. This modification enables the actuator to drive flexible electrodes with vibrational forward implantation.

Secondly, the clamp unit of the actuator should provide minimal clamp force (in the order of millinewtons) to avoid bending or rupture of the flexible electrode, and sufficient travel to ensure the complete release of the electrode. Minimal clamp force and sufficient travel ask for much lower stiffness of the DVIPA clamp unit than existing ones.

Lastly, previous studies indicate that vibration-assisted implantation requires speeds of 0.1–2 mm/s, vibration frequencies below 100 Hz (with optimal frequency at 60 Hz), and vibration amplitudes of 1–10 μm [25,26]. An approximate step displacement of 110 um, or rather 11× flexure hinge magnification for a 10 um travel piezoelectric stack, is required when the actuator works at a driving frequency of 20 Hz.

**Figure 1:**
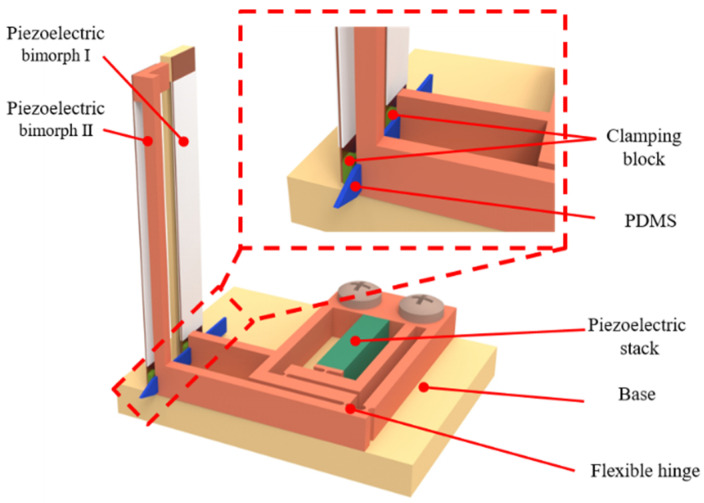
Structure of the proposed piezoelectric actuator.

To meet these requirements, a DVIPA is designed as shown in Figure 1. It primarily consists of a piezoelectric stack, two piezoelectric bimorphs, and flexible hinge. The piezoelectric stack provides the displacement and vibration required during the implantation of the flexible electrode, a function that has been previously discussed. The piezoelectric bimorphs serve as the main component of the clamping unit, as they can provide sufficient deformation displacement while also delivering an appropriate clamping force to prevent the flexible electrode from bending or breaking. The flexible hinges are used to amplify the small displacements generated by the piezoelectric stack, thus meeting the implantation requirements and ensuring more stable operation of the DVIPA.

### 2.2. Working Principle

**Figure 2:**
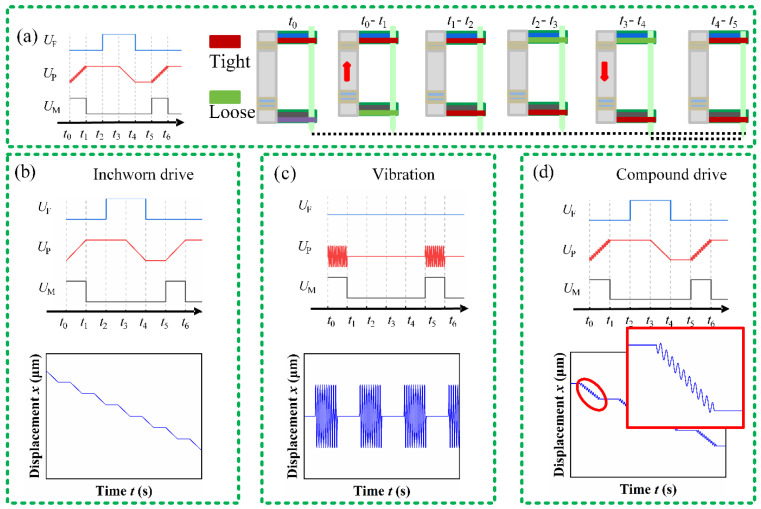
Drive–vibration composite scheme: (**a**) the principle of inchworm movement; (**b**) driving signal and displacement; (**c**) vibration signal and displacement; (**d**) composite signal and displacement.

The piezoelectric actuator described in this article operates in inchworm mode, as illustrated in Figure 2a. The movement during each cycle can be divided into six stages:

Initial State (*t*_0_): The movable clamp is activated; the fixed clamp releases. The driving hinge is in its initial state.

Stage I (*t*_0_ to *t*_1_): The exciting voltage of the piezoelectric stack(*U*_P_) increases. The piezoelectric stack vibrates and elongates, causing the driving hinge to transition to a stretched state, while the movable clamp drives the PDMS downward, resulting in a displacement of Δ*x*.

Stage II (*t*_1_ to *t*_2_): The exciting voltage of the fixed clamp(*U*_F_) decreases. The fixed clamp tightens.

Stage III (*t*_2_ to *t*_3_): The exciting voltage of the movable clamp(*U*_M_) decreases. The movable clamp releases.

Stage IV (*t*_3_ to *t*_4_): The *U*_P_ decreases. The piezoelectric stack shortens, causing the driving hinge to return to its initial state. The movable clamp moves upward to its original position, while the PDMS position remains unchanged.

Stage V (*t*_4_ to *t*_5_): The *U*_M_ increases. The movable clamp tightens.

Stage VI (*t*_5_ to *t*_6_): The *U*_F_ increases. The fixed clamp releases, and the actuator returns to its initial state.

Thus, the actuator proposed in this article can drive the PDMS to move downward by a distance Δ*x* in each complete movement cycle, which corresponds to the displacement Δ*x* output in stage I.

The actuator utilizes the piezoelectric stack as both the driving and vibrating power source, setting the control signal for stage I as a superposition of the driving signal and the vibrating signal, that is

(1) $$U_{p}=U_{d}+U_{V}$$

where *U*_p_ is the exciting voltage of the piezoelectric stack, *U*_d_ is the driving signal, and *U*_V_ is the vibration signal. Where *U*_d_ is a low-frequency trapezoidal wave providing forward displacement, and its function is:

(2) $$U_{d}\left(t\right)=\left\{\begin{array}{ll} \frac{A_{d}}{t_{rise}}t & 0\leq t<t_{\mathrm{rise}}\\ A_{d} & t_{\mathrm{rise}}\leq t<T-t_{\mathrm{fall}}\\ A_{d}-\frac{A_{d}}{t_{\mathrm{fall}}}\left(t-\left(T-t_{\mathrm{fall}}\right)\right) & T-t_{\mathrm{fall}}\leq t<T \end{array}\right.$$

where *t* is time, *T* is the period, *A*_d_ is the maximum amplitude of the trapezoidal wave, *t*_rise_ is the rise time, and *t*_fall_ is the fall time. Based on the aforementioned driving principles, when the piezoelectric stack is excited by *U*_d_, the PDMS outputs a step displacement as depicted in Figure 2b; *U*_V_ is a high-frequency sine wave used to provide vibration, and its function is as follows:

(3) $$U_{V}\left(t\right)=\left\{\begin{array}{ll} A_{V}sin\left(\omega t+\phi \right) & 0\leq t<t_{\mathrm{rise}}\\ 0 & t_{\mathrm{rise}}\leq t<T \end{array}\right.$$

where *A*_V_, ω and *ϕ* are the amplitude, angular frequency, and initial phase of the sine wave, respectively. When the piezoelectric stack is excited by *U*_V_, the PDMS outputs a high-frequency vibration as shown in Figure 2c. Combining (1), (2), and (3), the function of the composite waveform is:

(4) $$U_{P}\left(t\right)=\left\{\begin{array}{ll} \frac{A_{d}}{t_{rise}}t+A_{V}sin\left(\omega t+\phi \right) & 0\leq t<t_{\mathrm{rise}}\\ A_{d} & t_{\mathrm{rise}}\leq t<T-t_{\mathrm{fall}}\\ A_{d}-\frac{A_{d}}{t_{\mathrm{fall}}}\left(t-\left(T-t_{\mathrm{fall}}\right)\right) & T-t_{\mathrm{fall}}\leq t<T \end{array}\right.$$

when the piezoelectric stack is excited by *U*_P_, the PDMS generates a displacement with additional vibrations during forward movement, as illustrated in Figure 2d.

## 3. Modeling and Simulation

### 3.1. FEM Modeling

From the previous description, it is evident that the design of the flexure hinge significantly impacts the output performance of the actuator and determines the maximum average step displacement and amplitude of the actuator [27]. The maximum travel of the flexure hinge should be large enough to cover both the amplitude of vibration and the step displacement of the actuator. At the same time, increasing the single-step displacement can shorten the implantation time, which is beneficial for tissue recovery and recording quality [28]. A three-stage amplification flexure hinge with a right-circular flexure hinge design is proposed to achieve this. According to the literature [29], the compliance of the right-circular flexure hinge (*C*_R_) is calculated as follows:

(5) $$C_{R}=\left[\begin{array}{c} \begin{array}{cccccc} \frac{{du}_{x}}{{dF}_{x}} & 0 & 0 & 0 & 0 & 0\\ 0 & \frac{{du}_{y}}{{dF}_{y}} & 0 & 0 & 0 & \frac{{du}_{y}}{{dM}_{z}}\\ 0 & 0 & \frac{{du}_{z}}{{dF}_{z}} & 0 & \frac{{du}_{z}}{{dM}_{y}} & 0 \end{array}\\ \begin{array}{cccccc} 0 & 0 & 0 & \frac{{d\theta }_{x}}{{dM}_{x}} & 0 & 0\\ 0 & 0 & \frac{{d\theta }_{y}}{{dF}_{z}} & 0 & \frac{{d\theta }_{y}}{{dM}_{y}} & 0\\ 0 & \frac{{d\theta }_{z}}{{dF}_{y}} & 0 & 0 & 0 & \frac{{d\theta }_{z}}{{dM}_{z}} \end{array} \end{array}\right]$$

where *M*_x_, *M*_y_, *M*_z_ are the torques acting on the flexure hinge, while *F*_x_, *F*_y_, *F*_z_ are the applied forces. θ_x_, θ_y_, θ_z_, *u*_x_, *u*_y_, *u*_z_ are the rotations and deformations obtained in the three directions, respectively.

The calculation method for the rotational compliance of the right-circular flexure hinge around the *Z*-axis is as follows:

(6) $$\frac{{d\theta }_{z}}{{dM}_{z}}=\frac{12}{EdR^{2}}\left[\frac{2s^{3}\left(6s^{2}+4s+1\right)}{\left(2s+1\right){\left(4s+1\right)}^{2}}+\frac{12s^{4}\left(2s+1\right)}{{\left(4s+1\right)}^{5/2}}\tan^{-1}\sqrt{4s+1}\right]$$

where *R* is the radius of the right-circular flexure hinge; *E* is the elastic modulus; *d* is the width of the flexure hinge; and *s* is calculated as follows:

(7) s=t/R

where *t* is the thickness of the right-circular flexure hinge. Equations (5) and (6) are the equations for calculating the right-circular flexure hinge, and the output performance of the three-stage amplified flexure hinge used in this study can be computed based on these two equations. However, the three-stage amplified flexure hinge used here consists of more than ten right-circular flexure hinges, resulting in a very large computational workload. Therefore, this study employed COMSOL 6.1 finite element simulation to calculate the performance of the three-stage amplified hinge. The material used for the three-stage amplified flexure hinge is ASTM1045 steel, which is modeled as a linear elastic material in the simulation. The two holes on the base beam are completely fixed with no degrees of freedom. A fixed displacement of *L*_pzt_ = 10 μm was applied to simulate the input from the powered piezoelectric stack. A refined mesh was applied in the flexure hinge regions to ensure sufficient accuracy in stress and displacement analysis.

**Figure 3:**
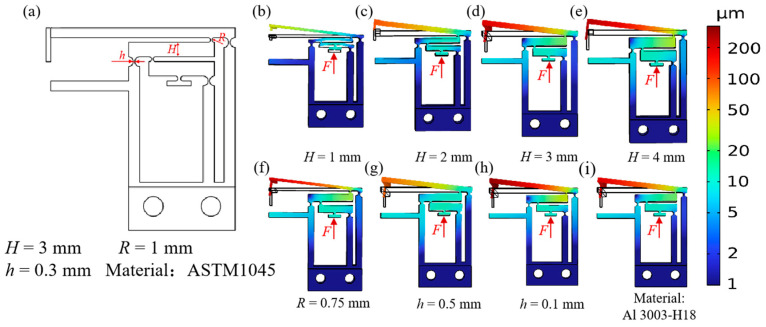
FEM statics analysis results. (**a**) Geometric parameters of the hinge (*h*, *R*, *H*); (**b**–**e**) simulation results with varying beam thickness (*H*); (f) simulation results with *R* = 0.75 mm; (**g**–**h**) simulation results with varying right-circular flexure hinge thickness (*h*); (**i**) simulation results with Al 3003-H18.

The finite element simulation results for the proposed flexure hinge are shown in Figure 3. Figure 3a illustrates the hinge parameters investigated through COMSOL simulations, including the right-circular flexure hinge thickness (*h*), the arc radius (*R*), and the beam thickness (*H*). The simulation results are shown in Figure 3b–g. The results indicate that the arc radius has minimal impact on the amplification factor of the hinge, whereas the thickness of the right-circular flexure hinge significantly affects it. Specifically, a smaller hinge thickness results in a higher amplification factor. Additionally, when the beam thickness is less than 3 mm, noticeable bending occurs, which reduces the amplification factor. For thicknesses greater than 3 mm, the impact becomes negligible. Taking into account both the amplification factor and manufacturing feasibility, this study determined the structural parameters of the flexure hinge. The final structural parameters are: *H* = 3 mm, *R* = 1 mm, *h* = 0.3 mm, and the material is ASTM 1045.

It should be noted that, although a minimum amplification ratio is required to achieve the target displacement, a higher amplification ratio is adopted in the design to provide sufficient margin under practical operating conditions.

### 3.2. Dynamical Modeling

**Figure 4:**
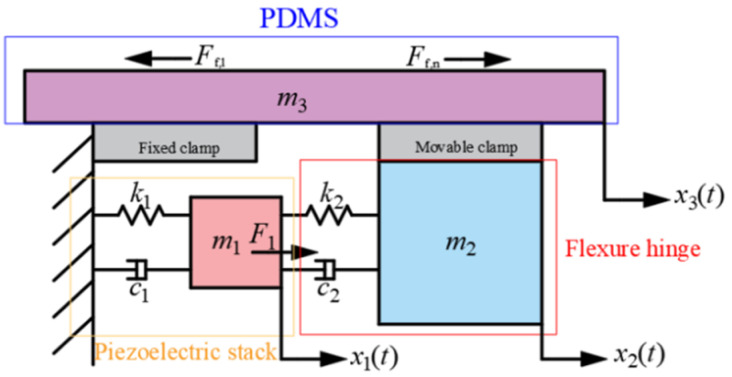
Schematic diagram of dynamic models.

Based on the analysis of the working principle illustrated in Figure 2, a dynamic model shown in Figure 4 was established. During Stage I, the piezoelectric stack undergoes elongation, and its dynamic differential equation is expressed as:

(8) $$m_{1}{\ddot{x}}_{1}+c_{1}{\dot{x}}_{1}+k_{1}x_{1}=F_{1}-F_{N1}-c_{2}\left({\dot{x}}_{1}-{\dot{x}}_{2}\right)-k_{2}\left(x_{1}-x_{2}\right)$$

(9) $$F_{1}=nd_{33}k_{1}U$$

where *m*_1_ is the equivalent mass of the stack, *c*_1_ is the damping coefficient, *k*_1_ is the stiffness coefficient, *F*_1_ is the piezoelectric driving force, *x*_1_ is the displacement of the piezoelectric stack, *F*_N1_ = *F*_N2_ is the contact force between the piezoelectric stack and the flexure hinge, *n* is the number of piezoelectric ceramic layers, *d*_33_ is the piezoelectric constant, and *U* is the voltage of the excitation signal.

The motion of the flexure hinge is influenced by the output force from the piezoelectric stack and its own elastic deformation. The dynamic relationship can be described as:

(10) $$m_{2}{\ddot{x}}_{2}+c_{2}{\dot{x}}_{2}+k_{2}x_{2}=F_{N2}+c_{2}\left({\dot{x}}_{1}-{\dot{x}}_{2}\right)+k_{2}\left(x_{1}-x_{2}\right)-F_{f,n}$$

where *m*_2_ is the equivalent mass of the flexure hinge, *c*_2_ is the damping coefficient, *k*_2_ is the stiffness coefficient, and *x*_2_ represents the displacement of the flexure hinge.

The motion of PDMS is directly coupled with the flexure hinge, and its dynamic equation is governed by the interaction force from the flexure hinge and PDMS’ inherent dynamic properties, expressed as:

(11) $$m_{3}{\ddot{x}}_{3}=F_{f,n}-F_{f,1}$$

where *m*_3_ is the equivalent mass of PDMS and *x*_3_ represents the displacement of PDMS. The displacement amplification characteristic of the flexure hinge, enabled by its lever-type amplification mechanism, results in an output displacement λ times greater than that of the piezoelectric stack. This relationship is mathematically expressed as:

(12) $$x_{2}=\lambda x_{1}$$

In conventional piezoelectric stepping actuators, the LuGre friction model is commonly employed to describe the relationship between actuator velocity and frictional forces. This model is based on the stiffness and damping characteristics of microscopic interfacial interactions (e.g., bristle-like asperities) at contact surfaces, allowing precise characterization of friction behavior under micrometer- to nanometer-scale displacements. The model can be expressed as:

(13) $$F_{f}=\sigma_{0}z+\sigma_{1}\dot{z}+\sigma_{2}v$$

(14) $$\dot{z}=v-\frac{\left|v\right|}{g(v)}z$$

(15) $$g\left(v\right)=F_{c}+\left(F_{s}-F_{c}\right)e^{{-(\frac{v}{v_{s}})}^{2}}$$

where z represents the average bristle deflection, v is the relative velocity, and $\sigma_{0}$, $\sigma_{1}$, and $\sigma_{2}$ denote the stiffness, damping, and viscous friction coefficients, respectively. $F_{s}$ and $F_{c}$ correspond to the static and Coulomb friction forces, and $v_{s}$ is the Stribeck velocity. The model parameters are selected based on representative values reported in the literature [30], which have been widely used for describing friction behavior in similar piezoelectric actuation systems.

**Figure 5:**
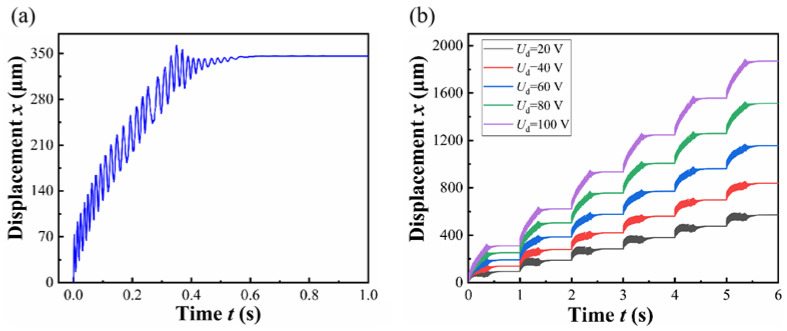
Displacement curve of simulation.

Based on the aforementioned dynamic equations, a comprehensive simulation model of the actuator was implemented in Simulink. The parameter values utilized in the Matlab 2022a/Simulink computations are systematically summarized in Table 1. Through numerical simulations, the actuator’s stepping gait was successfully characterized, with representative results presented in Figure 5a. The simulation results demonstrate that the actuator effectively achieves a hybrid motion mode combining continuous feeding with superimposed vibration. Furthermore, stepping response curves under varying driving voltages were systematically investigated, as depicted in Figure 5b. Quantitative analysis reveals that the total displacement per six operational cycles exhibits a positive correlation with the driving voltage, while the vibration amplitude remains voltage-independent.

## 4. Experiment

### 4.1. Experimental System

**Figure 6:**
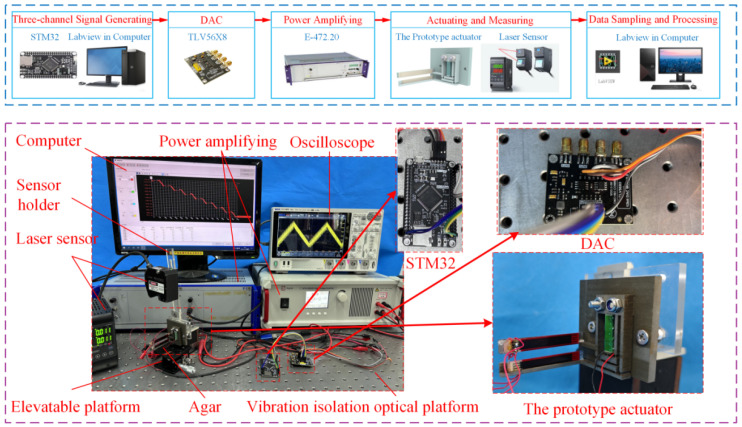
The setup of the experimental system.

A prototype of the DVIPA has been developed, and an experimental system has been set up, as shown in Figure 6. The experimental system mainly consists of a vibration isolation optical platform, a microcontroller unit (MCU), a digital-to-analog converter (DAC), two power amplifiers, an oscilloscope, a laser sensor, a personal computer (PC), and the prototype actuator. The entire experimental system is placed on a vibration-isolation optical platform.

The MCU (Alientek, STM32f407VET6, China) controls the DAC (TLV56X8) to generate three-channel signals which are amplified by power amplifiers (Physik Instrumente, E-472.20, Germany) to drive the prototype actuator. Meanwhile, the displacement generated by the PDMS is captured by a laser displacement sensor (KEYENCE, LK-H080, Japan) and then transmitted and recorded to the PC. Thus, the motion performance of the prototype can be obtained.

### 4.2. Composite Effect Testing

**Figure 7:**
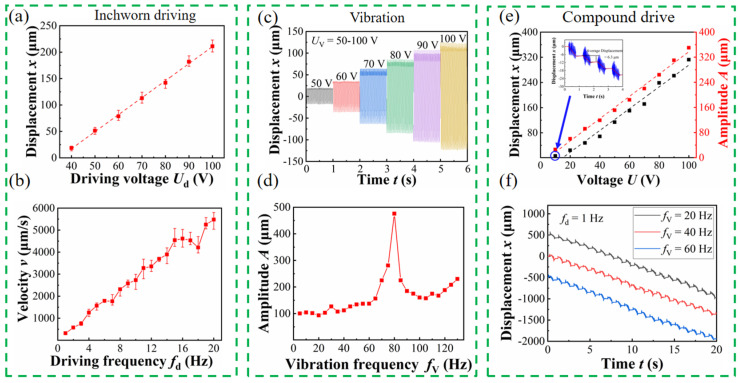
(**a**) Average step displacement under different driving voltages; (**b**) the velocity under different driving frequencies; (**c**) displacement history at different vibration voltages; (**d**) the amplitude at different vibration frequencies; (**e**) the effect of voltage under composite motion; (**f**) the effect of frequency under composite motion.

Figure 7a illustrates the relationship between the average step displacement of the prototype and the voltage when it works in inchworm drive mode, indicating a nearly linear relationship. When the driving voltage is set to 100 V, the average step displacement can reach 211 μm, providing sufficient travel margin for additional vibrations. Figure 7b presents the relationship between the prototype’s speed and frequency in inchworm drive mode. It can be seen that when the driving frequency is less than 15 Hz, the speed is positively correlated with the frequency, suggesting that the motion of the prototype is relatively stable at the required driving frequency. Figure 7c shows the amplitude of the prototype under different vibration signals. The figure indicates that the amplitude of the prototype linearly increases with the rise in voltage, allowing for convenient and intuitive amplitude adjustments.

Figure 7d illustrates the relationship between the amplitude of the prototype’s vibration and the vibration frequency. It shows that the first-order characteristic frequency of the prototype is 80 Hz, which is far from the required vibration frequency, allowing the prototype to maintain stability during operation. The voltage characteristic under the composite working state of drive and vibration is presented in Figure 7e, which shows that the composite operation has a minimal impact on the driving and vibration performance of the prototype. The vibration and driving work complement each other by compensating for the tolerances present during installation. Therefore, the displacement in the composite motion is greater than that in the individual driving or vibration modes. When the driving voltage is 10 V, the minimum step displacement of the actuator is 6.3 μm. Figure 7f also demonstrates the stepping effect of different frequency vibrations combined with the same driving signal. In conclusion, the prototype can achieve any form of composite vibration drive with a frequency range of 0–60 Hz and an amplitude range of 0–200 μm by adjusting the voltage frequency.

### 4.3. Stepping Characteristic

The displacement history testing aims to verify the motion mode of the integrated piezoelectric actuator designed for vibration-assisted flexible electrode implantation. Under a vibration frequency of 50 Hz, a driving frequency of 1 Hz, and no-load conditions, the experiment adjusts the driving and vibration voltage for testing.

**Figure 8:**
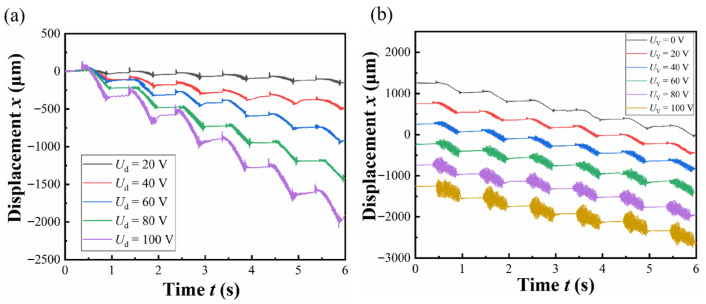
(**a**) Displacement history at different vibration voltages; (**b**) displacement history at different driving voltages.

Figure 8a shows the displacement history at a driving frequency of 1 Hz, a driving voltage of 100 V, and varying vibration voltages; Figure 8b illustrates the displacement history at a driving frequency of 1 Hz, a vibration voltage of 20 V, and different driving voltages. This experiment demonstrates that the prototype can achieve a regular and stable composite vibration driving motion mode at any driving and vibration voltages within the operational range. Additionally, the vibration and driving functions are relatively independent, meaning that changing one piezoelectric parameter does not affect the performance of the other, making it very convenient to adjust the actuator’s motion parameters.

### 4.4. Repeatability

**Figure 9:**
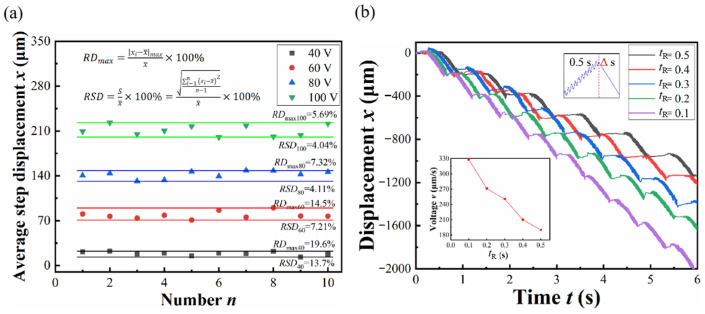
(**a**) Repetitive experiment with different driving voltages; (**b**) displacement history at different driving recovery times of the piezoelectric stack (*t*_R_).

Repeatability is an important indicator for evaluating the stability of piezoelectric actuators. The maximum relative deviation and the relative standard deviation are chosen as two metrics to verify the repeatability of the proposed actuator. The maximum relative deviation (*RD*_max_) is calculated using (16):

(16) $${RD}_{max}=\frac{{\left|x_{i}-\bar{x}\right|}_{max}}{\bar{x}}\times 100\%$$

where *x*_i_ is the average step displacement of the *i*th experiment, and $\bar{x}$ is the average step displacement of *N* experiment times. The relative standard deviation (*RSD*) is calculated using (17):

(17) $$RSD=\frac{S}{\bar{x}}\times 100\%=\frac{\sqrt{\frac{\sum_{i-1}^{n}{\left(x_{i}-\bar{x}\right)}^{2}}{n-1}}}{\bar{x}}\times 100\%$$

where *S* is the standard deviation and *N* is the number of repetitions. The experiments were conducted at a vibration signal of 50 Hz and 20 V, with a driving frequency of 1 Hz, using driving voltages of 40 V, 60 V, 80 V, and 100 V, repeated 10 times. As shown in Figure 9a, the *RD*_max_ of the average step displacement is less than 20% and the *RSD* is less than 15% under any voltage. At 100 V, the relative standard deviation is as low as 4.04%. This demonstrates that the actuator is relatively stable and exhibits good repeatability.

### 4.5. Recovery Time and Implant Performance

To reduce the implantation time without affecting the instantaneous speed of PDMS when implanted into brain tissue, a method was proposed that involves changing the recovery time of the piezoelectric stack (*t*_R_). The experimental fixed elongation time of the piezoelectric stack was set to 0.5 s, while the recovery time was set to 0.5, 0.4, 0.3, 0.2, and 0.1 s. The motion patterns and speeds of the actuator were tested under a driving voltage of 100 V and a vibration signal of 20 V at 50 Hz. The experimental results are shown in Figure 9b. The data indicate that altering the recovery time of the piezoelectric stack does not affect the instantaneous speed of PDMS implantation. Simultaneously, reducing the recovery time from 0.5 s to 0.1 s increased the average speed of PDMS implantation from 190 μm/s to 328 μm/s, significantly enhancing the implantation efficiency.

In order to simulate the impact of brain tissue resistance on the implantation performance of PDMS, implantation experiments were designed. Based on the literature review, agarose gels of certain concentrations have been widely used as mammal brain tissue models, specifically gel at 0.6% in DI water.

Regarding the implanted object, commercial finished flexible neural electrodes are complex to fabricate and relatively expensive. Typically, a flexible neural electrode consists of a polymer substrate and a nanoscale metallic conductive layer. Since the Young’s modulus of brain tissue is extremely low (typically in the range of 1–10 kPa), traditional rigid silicon or metal electrodes (100–200 GPa) often cause a severe mechanical mismatch. PDMS has been widely adopted as an ideal flexible substrate because its Young’s modulus is in the order of 1–3 MPa, which is orders of magnitude lower than rigid materials, thereby significantly reducing the mechanical mismatch with soft neural tissues [31]. Furthermore, because the thickness of the metal layer is negligible compared to the substrate, the overall mechanical behaviors of the electrode—such as bending stiffness and surface friction—are predominantly governed by the PDMS substrate [32]. Therefore, employing commercial PDMS cantilevers serves as a highly reliable and mechanically equivalent substitute for finished flexible electrodes to validate the vibration-assisted implantation process.

**Figure 10:**
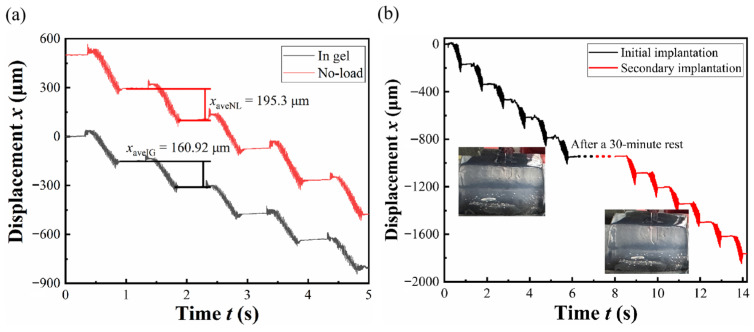
(**a**) The output displacement characteristics under unload and implantation conditions; (**b**) displacement history of initial implantation and secondary implantation.

A prototype was utilized to implant PDMS into the agarose gels, and its displacement was measured and compared with the displacement under no-load. The experimental results are shown in Figure 10a. The data indicate that the implantation reduces the actuator’s speed to some extent but minimally impacts the actuator’s performance.

The ability to perform re-insertion after initial implantation is considered a potential advantage of vibration-assisted implantation. In this study, a secondary insertion experiment was conducted to preliminarily evaluate this capability. The experiment was performed at a vibration frequency of 50 Hz and a voltage of 50 V. The results are shown in Figure 10b, where the black curve represents the displacement during the initial insertion, and the red curve corresponds to the secondary insertion after a 30 min interval. The results indicate that the prototype is capable of repositioning the PDMS after an initial insertion and a resting period. It should be noted that this experiment is conducted in an agarose gel model and is intended as a proof-of-concept demonstration. Further validation under more realistic biological conditions is required in future work.

**Figure 11:**
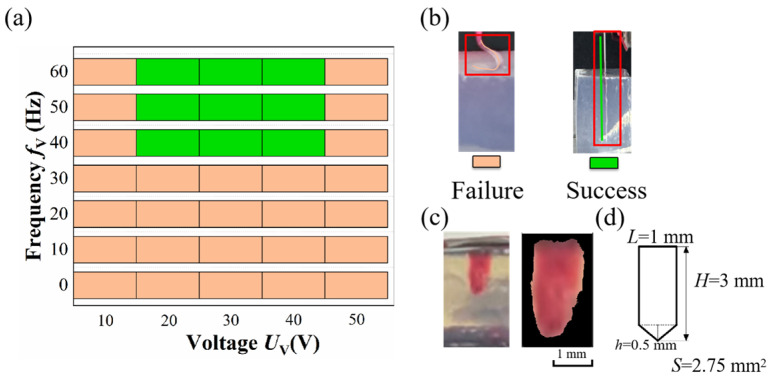
(**a**) The impact of vibration on PDMS implantation; (**b**) PDMS morphology of buckling failure and successful implantation; (**c**) the digital image of damage area; (**d**) the dimensions of the PDMS.

A prototype was used to drive PDMS into agarose gels to verify the effect of vibration-assisted flexible electrode implantation. The experiment tested whether different vibration voltages and frequencies could assist in the effective implantation of a PDMS cantilever (3 mm in effective unsupported length, 1 mm in width, and 100 μm in thickness) into the agarose gels under a driving voltage of 100 V. The results are shown in Figure 11a. Experimental results indicate that vibrations with frequencies above 40 Hz and voltages between 20 and 40 V can effectively aid in the implantation of PDMS into agarose gels. Under vibration assistance, the implantation form of PDMS changed from the buckling failure state to the effective implantation state, as shown in Figure 11b. This experiment demonstrates that the prototype can help PDMS implant into agarose gels through vibration-assisted methods.

For damage evaluation, 0.6% agarose gel was used as a visualizable medium, and red dye was introduced to highlight the damaged region. After implantation, images were captured using a fixed-position camera at a known distance, as shown in Figure 11c. To quantify the implantation-induced damage, a color-based image processing method was applied. The images were converted into HSV (or LAB) color space, followed by threshold segmentation based on the hue and saturation distribution of the red region. Morphological operations were used to remove noise and obtain a continuous damage region. The pixel area was then converted into a physical area using a calibration factor determined from the known dimensions of the PDMS in the image. Each image was processed independently five times, and the extracted red pixel area was recorded for each trial. The final damage area was reported as the mean value with standard deviation, providing an estimate of the measurement variability and method stability.

The resolution of the images was approximately 9.4 μm/pixel. Each sample was processed five times, and the damage area was reported as the mean value with standard deviation. As shown in Figure 11c, the extracted damaged region corresponds to an area of approximately 3.39 mm^2^, while the cross-sectional area of the PDMS is about 2.75 mm^2^ (Figure 11d). These results indicate that the implantation process leads to an increase in the affected area of approximately 23%.

## 5. Discussion

This study extends the design focus of piezoelectric actuators, to some extent, from solely pursuing extreme actuation performance toward functional integration for flexible electrode implantation tasks.

In terms of actuation capability, the proposed DVIPA achieves a minimum step size of 6.4 μm and a maximum velocity of 328 μm/s. For flexible neural electrode implantation, positioning requirements are typically on the micrometer scale, considering that neural structures generally span tens to hundreds of micrometers. From this perspective, the achieved resolution and speed are sufficient to support controlled insertion processes. Consequently, further improvement in these metrics may not be the primary factor limiting system performance in the targeted application.

In contrast to conventional approaches that rely on separate driving and vibration modules, the proposed actuator integrates both functions within a single structure by employing a shared piezoelectric stack and a composite excitation strategy. This configuration enables simultaneous generation of stepping motion and vibration, while reducing system complexity and allowing flexible adjustment of operating parameters.

To further contextualize the proposed approach, a qualitative comparison with representative neural electrode implantation systems is presented in Table 2.

As indicated, the DVIPA demonstrates a relatively higher level of integration by avoiding additional vibration modules and auxiliary structures. Such a feature may be beneficial in space-constrained scenarios, including stereotaxic implantation setups.

However, this integrated design also introduces certain trade-offs. Achieving sufficient step displacement requires a relatively high amplification ratio in the flexure hinge, which may reduce structural stiffness and influence the effective bandwidth of the actuator. Therefore, a balance between displacement amplification and dynamic performance is necessary in practical implementations.

Overall, the proposed DVIPA emphasizes structural integration and functional coupling while maintaining actuation performance at a level suitable for current implantation requirements. Future work will focus on optimizing the flexure hinge design to improve the balance between amplification and dynamic characteristics, as well as validating the system under more representative experimental conditions.

## 6. Conclusions

A DVIPA for flexible electrode vibration-assisted implantation is proposed. It enables independent implantation of flexible electrodes without in vivo assistance, allowing for real-time signal acquisition, minimizing damage, and meeting the need for secondary implantation. The piezoelectric stack was utilized as power sources for both drive and vibration. A composite waveform of low-frequency trapezoidal waves and high-frequency sine waves is used as the excitation signal for the piezoelectric stack, achieving a compound mode of drive and vibration. A prototype of DVIPA was constructed, and the output performance was tested. Conclusions could be made from the experimental results:

(1) The DVIPA, excited by the drive–vibration composite waveform, can generate additional vibrations to inchworm movement.
(2) The output vibration and inchworm motion of DVIPA can be adjusted by voltage and frequency independently.
(3) The minimum step displacement of the prototype can reach 6.3 μm, and its speed can reach 328 μm/s. The vibration is adjustable within a frequency range of 0–60 Hz and an amplitude range of 0–200 μm.
(4) Vibrations with frequencies above 40 Hz and voltages between 20 and 40 V can effectively assist PDMS implantation into agarose gels. The implantation damage area increased by only 23% compared to the implanted PDMS by the prototype.
(5) The DVIPA can carry out secondary implantation without additional aids when glial encapsulation occurs.

## Figures and Tables

**Table 1:** Parameters and their value in Simulink.

Parameter	Value	Parameter	Value
*m*_1_	0.75 × 10^−3^ kg	*n*	130
*m*_2_	12 × 10^−3^ kg	d_33_	720 × 10^−12^ m·V^−1^
*m*_3_	4.2 × 10^−3^ kg	*λ*	20
*c*_1_	40 N·s·m^−1^	*c*_2_	180 N·s·m^−1^
*k*_1_	17 × 10^6^ N·m^−1^	*k*_2_	5 × 10^5^ N·m^−1^

**Table 2:** Qualitative comparison of neural electrode implantation systems.

Reference	Drive Mechanism	Overhang Strategy	Assistance Strategy	System Integration
Vitale et al. [13]	Fluidic	Dynamic (fluid support)	Microfluidic channel	Low
Hanson et al. [33]	Motorized drive	Fixed (base-clamping)	Stiff needle	Low
Chen et al. [34]	Independent piezo + vibration	Fixed (base-clamping)	No	Medium
Li et al. [35]	Piezoelectric drive	Dynamic (stepping)	No	Medium
This work (DVIPA)	Integrated piezo drive–vibration	Dynamic (stepping)	Intrinsic vibration assistance	High

## Data Availability

The data presented in this study are available on request from the corresponding authors.
